# Notable impact of wildfires in the western United States on weather hazards in the central United States

**DOI:** 10.1073/pnas.2207329119

**Published:** 2022-10-17

**Authors:** Yuwei Zhang, Jiwen Fan, Manish Shrivastava, Cameron R. Homeyer, Yuan Wang, John H. Seinfeld

**Affiliations:** ^a^Atmospheric Science and Global Change Division, Pacific Northwest National Laboratory, Richland, WA 99352;; ^b^School of Meteorology, University of Oklahoma, Norman, OK 73072;; ^c^Division of Geological and Planetary Sciences, California Institute of Technology, Pasadena, CA 91125;; ^d^Division of Chemistry and Chemical Engineering, California Institute of Technology, Pasadena, CA 91125

**Keywords:** wildfire, severe convective storm, hail, extreme precipitation, biomass burning aerosols

## Abstract

Increased wildfire events constitute a significant threat to life and property in the United States. Wildfire impact on severe storms and weather hazards is another pathway that threatens society, and our understanding of which is very limited. Here, we use unique modeling developments to explore the effects of wildfires in the western US (mainly California and Oregon) on precipitation and hail in the central US. We find that the western US wildfires notably increase the occurrences of heavy precipitation rates by 38% and significant severe hail (≥2 in.) by 34% in the central United States. Both heat and aerosols from wildfires play an important role. By enhancing surface high pressure and increasing westerly and southwesterly winds, wildfires in the western United States produce (1) stronger moisture and aerosol transport to the central United States and (2) larger wind shear and storm-relative helicity in the central United States. Both the meteorological environment more conducive to severe convective storms and increased aerosols contribute to the enhancements of heavy precipitation rates and large hail. Moreover, the local wildfires in the central US also enhance the severity of storms, but their impact is notably smaller than the impact of remote wildfires in California and Oregon because of the lessened severity of the local wildfires. As wildfires are projected to be more frequent and severe in a warmer climate, the influence of wildfires on severe weather in downwind regions may become increasingly important.

## Significance

Wildfires have intensified in both frequency and burned areas in recent decades in the United States and constitute a significant threat to life and property. Sensible heat and aerosols produced by wildfires may affect severe storms and weather hazards downstream. Here, we show that wildfires in the western United States can lead to more severe hazardous weather in the central United States, notably increasing occurrences of heavy precipitation rates and large hail. Both heat and aerosols from wildfires play an important role. As wildfires are projected to be more severe in a warmer climate, the influence of wildfires on severe weather in downstream regions may become increasingly important.

The frequency and burned area of forest wildfires have increased globally in recent decades (1, 2), although the total burned area around the globe declined primarily due to agricultural expansion and intensification (3). In the western United States (WUS), large wildfires have become more frequent and have emerged as a significant threat to health, life, and property (1, 4). Likewise, severe convective storms and their associated hazards (e.g., hail, tornado, lightning, flood) cause significant property damage and economic losses in the United States (5). By influencing severe convective storms, wildfires pose a further threat to life and property. Wildfires can affect severe convective storms and weather by releasing large amounts of aerosol particles (6) and sensible heat (7). The former can affect storm intensity and weather hazards through aerosol–cloud interactions (8–11). The latter modifies the environmental thermodynamics and can generate pyrocumulonimbus (pyroCb) clouds (12–17).

Studies of wildfire effects on severe convective storms and weather hazards focused on the pyroCb (7, 12–17) or local wildfire aerosol effects (8–11). Few studies have explored the impact of long-range transported biomass burning aerosols on severe storms (18–20). There is a lack of studies about the effects of wildfire on remote storms through meteorological changes generated by wildfires. In the WUS, large wildfires emit enormous quantities of aerosols and sensible heat during wildfire seasons. The environment in the central United States (CUS) can be affected by the wildfires in the WUS through transported aerosol and gas-phase pollutants. In addition, WUS wildfires heat the environment, potentially perturbing synoptic-scale meteorology, especially when the burned area is large, with an extended burning period (21, 22).

**Fig. 1.**
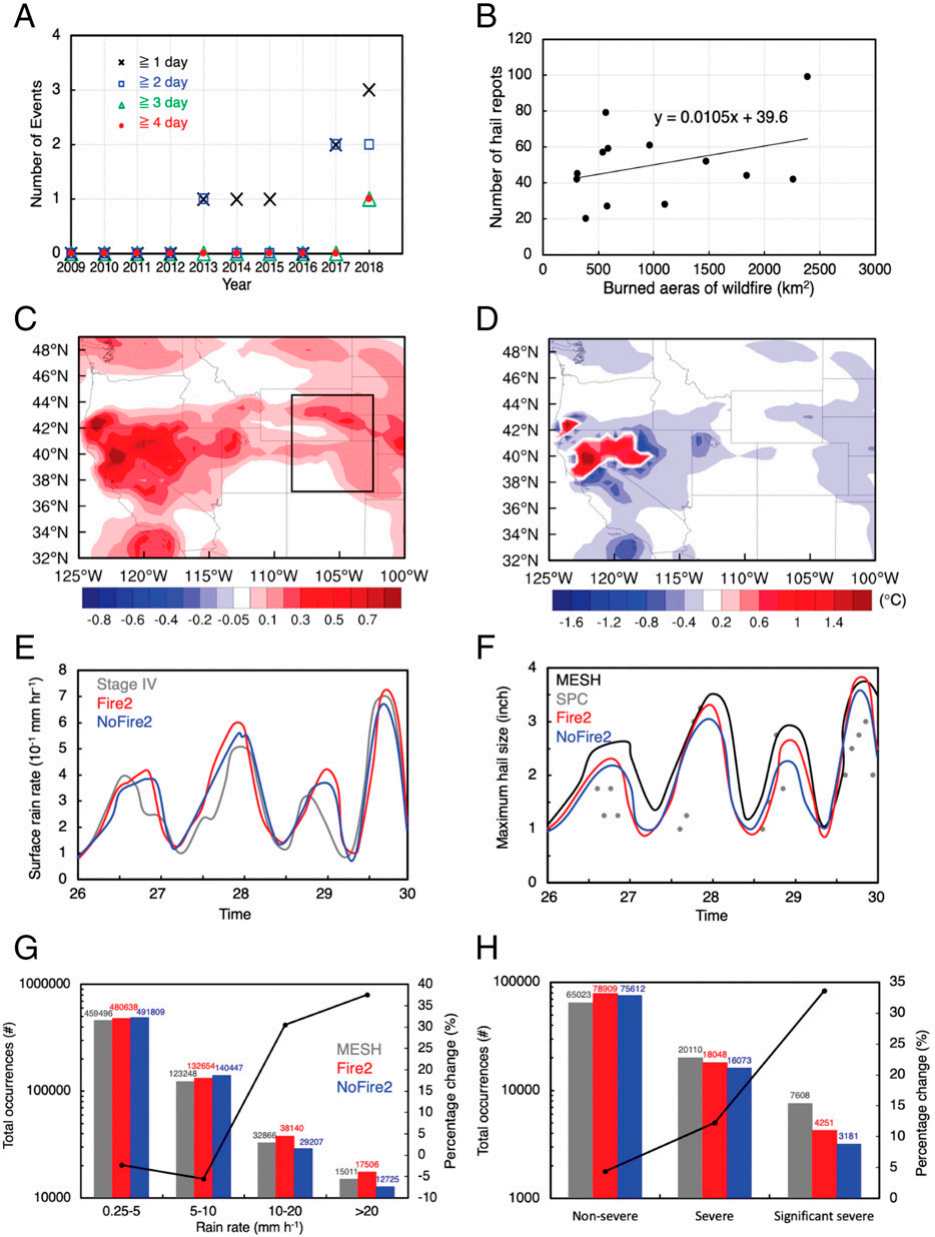
(*A*) Observed co-occurrences of storms in the CUS with wildfires in the WUS over 2009 to 2018, with different colors for different storm days (e.g., red is for the storm events occurring on ≥4 consecutive days). (*B*) Observed correlation of the number of daily hail reports in the CUS with the burned area of the WUS wildfires for the co-occurring events with storms occurring on ≥2 consecutive days. The burned area shown here is summed over 3 d (i.e., the current storm day plus the 2 d before the storm) to account for the time lag of the WUS wildfire effects. Differences in (*C*) AOD and (*D*) 2-m temperature averaged over July 26 to 29 (excluding the storm periods defined in Materials and Methods) between Fire1 and NoFire1. Time series of (*E*) surface rain rate and (*F*) maximum hail size from observations (gray or black), Fire2 (red), and NoFire2 (blue) at the storm region during the storm periods. Occurrences for (*G*) rain rates of 0.25 to 5, 5 to 10, 10 to 20, and >20 mm h^−1^ and (*H*) nonsevere (0.5 cm ≤ diameter < 2.5 cm), severe (2.5 cm ≤ diameter < 5 cm), and significant severe hail (SSH; ≥5 cm) from observation (gray), Fire2 (red), and NoFire2 (blue) for the storm region during the storm periods. The black line in (*G*) and (*H*) denotes the percentage change (secondary y axis) from NoFire2 to Fire2. The black box in (*C*) marks the study area for precipitation and hail. See Materials and Methods for the description of the observational datasets stage IV, MESH, and SPC.

It has been thought that WUS wildfire seasons (fall and winter) do not overlap with CUS severe weather seasons (spring and summer). However, as seen in recent years, the wildfire season is starting progressively earlier (23); in 2018, the wildfire season started in May in both the WUS and CUS. Therefore, the co-occurrence of WUS wildfires with CUS severe weather appeared. We analyzed observed co-occurrences of storms with hail and heavy precipitation reports in the CUS states and wildfires in the WUS states over 10 y, from 2009 to 2018 (see *Materials and Methods*). Co-occurring events with storms on 2 consecutive days are found only in 2013, 2017, and 2018 (Fig. 1 *A*, blue). Co-occurring events with storms on 3 or 4 consecutive days happened only in 2018 (Fig. 1 *A*, green and red). Therefore, here, we address the extreme phenomenon that it is reasonable to expect to occur more frequently in the future as both the wildfire potential in the WUS and severe weather in the CUS in summer are projected to be increased based on high-resolution simulations (24, 25).

Although the co-occurrences are rare and statistically significant observational analysis cannot be achieved, we still analyzed the correlation of the number of daily hail reports in the CUS with the burned area of WUS wildfires for the co-occurring events with storms on ≥2 consecutive days as identified in Fig. 1*A*. A positive correlation is found (Fig. 1*B*), which is encouraging in the study of a potential connection between the WUS wildfires and the CUS severe weather. In addition to the potentially remote effects of wildfires in the WUS, storms in CUS states such as Colorado and Wyoming could be affected by local wildfires in the Rocky Mountains. When wildfires occur simultaneously in both the WUS and CUS, severe convective storms and weather hazards in the CUS could be jointly affected by both remote wildfires in the WUS and local wildfires. Here we address the questions below: What are the joint and respective effects of remote and local wildfires and the major mechanisms by which wildfires affect the CUS severe weather? Are the effects mainly contributed by the released sensible heat or aerosol or both?

We have addressed these questions mainly through real-case simulations at convection-permitting scales using unique modeling tools in conjunction with field measurements of aerosol, meteorology, fire, clouds, precipitation, and hail for an extreme co-occurring event with storms occurring on 4 consecutive days in 2018 (the only one from 1999 to 2018 based on our examination). During the week of July 23 to 29, 2018, four severe convective storm events occurred on each day from July 26 to 29 in Colorado, Wyoming, Nebraska, and South Dakota. Significant wildfire events occurred in California and Oregon at the same time and before the first storm occurred (see *Materials and Methods*). Those storms were spawned by a stalled weather pattern—a weak surface low and a frontal boundary, producing flash flooding, large hail (2.75 in. [baseball size]), straight-line winds gusting above 90 mph, and several tornado touchdowns (26). Total economic and insured losses caused by those storms were expected to exceed $100 million (26).

To realistically simulate deep convective clouds and aerosols, we carried out high-resolution simulations by using the chemistry version of the Weather Research and Forecasting model (WRF-Chem) coupled with a spectral-bin microphysics scheme (SBM), which is a benchmark model for aerosol–cloud interaction studies (27). The model has demonstrated good performance in simulating deep convective storms (28–30). Moreover, to account for the effect of sensible heat flux from wildfires, we incorporated the parameterization of sensible heat from wildfires as developed by Zhang et al. (16) into WRF-Chem-SBM. We used a “nest down” approach, with two nested domains of horizontal grid spacings of 3 and 1 km (*SI Appendix*, Fig. S1*A*), with domain 1 (D01) covering both the WUS and CUS and domain 2 (D02) focusing on the CUS storm simulation. With WRF-Chem, both aerosol–radiation interactions and aerosol–cloud interactions were considered.

The model simulations of the extreme co-occurring event are listed in Table 1, for which a detailed description is presented in *Materials and Methods*. Briefly, Fire1 and NoFire1 are the simulations covering both the WUS and CUS, with wildfire effects considered and excluded, respectively. Several simulations were carried out over the storm region (mainly Colorado, Wyoming, Nebraska, and South Dakota). The difference between Fire2 and NoFire2 is the total wildfire effect on severe storms. We separated contributions from the remote wildfire effect and the local wildfire effect. The remote wildfire effect is the difference between Fire2 and Fire2L, and the local wildfire effect is the difference between Fire2 and Fire2R. The wildfire heat and aerosol effects were also examined based on the sensitivity tests Fire2_NH and Fire2_NRH. For the event studied, the WUS refers to the region principally in Oregon and California, and the CUS mainly refers to Colorado, Wyoming, Nebraska, and South Dakota.

**Table 1.** Description of model simulations

Simulation for domain 1	
Fire1	Simulation with wildfires considered.
NoFire1	Simulation without wildfires considered.
Fire1_NH	Based on Fire1, turn off the heat effect of wildfires.
Simulation for domain 2	
Fire2	Simulation with both local and remote wildfire effects considered, using the initial and boundary conditions in meteorology and aerosols from Fire1.
NoFire2	Simulation without wildfires (both local and remote wildfire effects are excluded), using the initial and boundary conditions in meteorology and aerosols from NoFire1.
Fire2R	Based on Fire2, consider the remote wildfire effect only (local wildfires are turned off).
Fire2L	Based on Fire2, consider the local wildfire effect only (the effects of remote wildfires are excluded—in other words, using the initial and boundary conditions in meteorology and aerosols from NoFire1).
Fire2_NH	Based on Fire2, consider the wildfire aerosol effect only (the heat effect from both remote and local wildfires is excluded—in other words, using the initial and boundary conditions in meteorology and aerosols from Fire1_NH and turning off the heat effect from local wildfires).
Fire2_NRH	Based on Fire2, exclude the heat effect from the remote wildfires (i.e., using the initial and boundary conditions in meteorology and aerosols from Fire1_NH).

## Results

### Wildfires Enhance Occurrences of Heavy Precipitation and Large Hail.

The model performance is comprehensively evaluated for various aspects: aerosols, meteorology, fire, precipitation, and hail. The simulated aerosols, fire plume height, and meteorology over the WUS and CUS are evaluated first. The baseline simulation over the WUS and CUS, Fire1, in which both remote and local wildfire effects are considered, captures the location and intensity of aerosol optical depth (AOD) observed by the Moderate Resolution Imaging Spectroradiometer (MODIS) from July 26 to 29 (*SI Appendix*, Fig. S1). Aerosols produced in the WUS are transported to the CUS (Fig. 1*C* and *SI Appendix*, Fig. S1). On average, the wildfires led to an increase in AOD by up to 0.9 in the WUS (e.g., California, Nevada) and 0.4 in the CUS over July 26 to 29. Also, Fire1 well simulates the observed particulate matter (PM2.5) concentration on the surface from the Environmental Protection Agency (EPA). There are certain values in the wildfire center in which observations either were absent or had large measurement errors (*SI Appendix*, Fig. S2*B*). The surface PM2.5 concentrations do not show a strong transport of aerosols from the WUS as in the AOD plots. This is because the transport of aerosols mainly occurs at elevated levels (high PM2.5 concentration peaks at ∼2.5 km, as shown in *SI Appendix*, Fig. S2*C*). The plume heights for the wildfires over California are well simulated by Fire1 (*SI Appendix*, Fig. S2*D*), demonstrating a good agreement with the retrieved data from the Multiangle Imaging SpectroRadiometer (MISR). The observed low-level temperature and humidity are also captured (*SI Appendix*, Figs. S3 and S4). Wildfires increased the 2-m temperature by up to 2.4 °C averaged over July 26 to 29 due to released sensible heat (the maximal increase averaged over the fire area during the period is 4.8 °C), but decreased the temperature over a broad area of the WUS and CUS by 0.2 to 0.6 °C (Fig. 1*D*). This is a result of the reduction in solar radiation by increased aerosol scattering or absorption.

Regarding the simulated storm properties, Fire2 represents the baseline simulation of the observed storms. The temporal evolution of precipitation rates and hail occurrences over the storm region (black box in Fig. 1*C*) is simulated reasonably well (Fig. 1 *E* and *F*), with an overprediction of precipitation rates on July 28. The simulation reproduces the frequency distributions of precipitation rates and hail over different categories (red versus gray in Fig. 1 *G* and *H*). The joint effect of both WUS and CUS wildfires increases the occurrences of heavy precipitation rates (>20 mm h^−1^) and significant severe hail (SSH; >2 in. in diameter) by 38% and 34%, respectively (Fig. 1 *G* and *H*, secondary axis). The accumulated rainfall over July 26 to 29 increases by ∼5.9 mm (19%). Moreover, wildfires decrease occurrences of light (0.25 to 5 mm h^−1^) and moderate (5 to 10 mm h^−1^) rain but enhance occurrences of heavy precipitation rates and large hail. Indeed, the convective intensity is notably enhanced by wildfires with increased frequencies of strong updrafts (>10 m s^−1^) and correspondingly reduced frequencies of weak updraft velocities (<5 m s^−1^), as shown in *SI Appendix*, Fig. S5 *A* and *B*. The maximum updraft speed with a frequency exceeding 0.1% is increased from 26 to 34 m s^−1^ (by ∼30%).

### Relative Significance of Remote and Local Wildfire Effects.

**Fig. 2.**
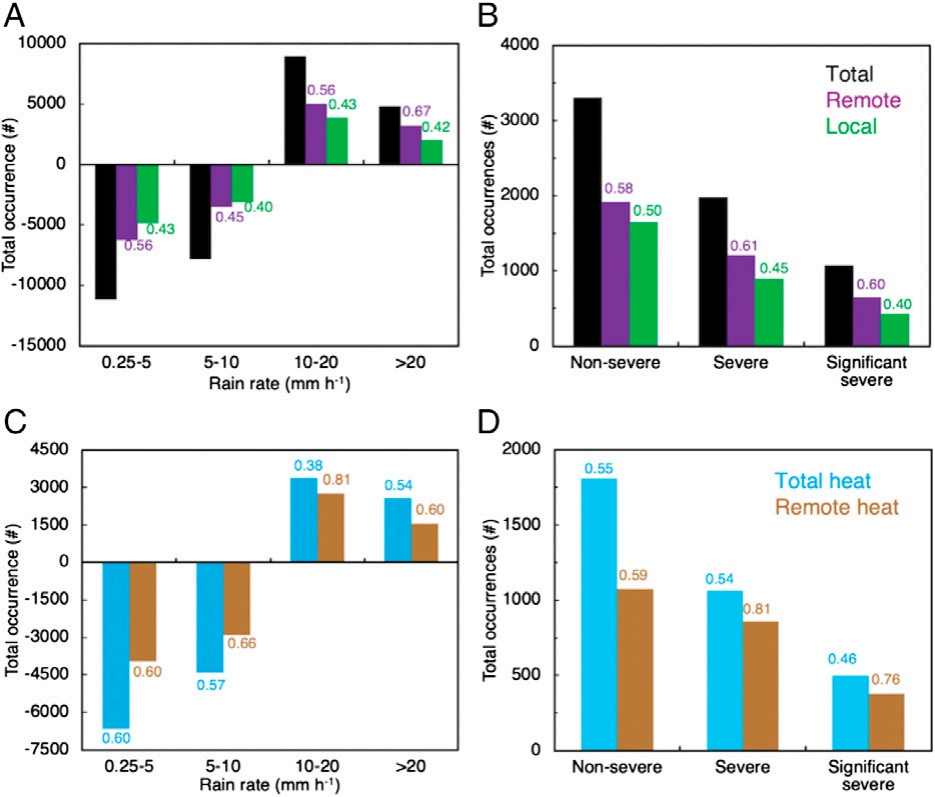
Differences in occurrence of (*A*) rain rates of 0.25 to 5, 5 to 10, 10 to 20, and >20 mm h^−1^ and (*B*) nonsevere (0.2 ≤ diameter < 1 in.), severe (1 ≤ diameter < 2 in.), and SSH (≥2.5 in.) due to total wildfire effect (black; Fire2-NoFire2), remote wildfire effect (purple; Fire2-Fire2L), and local wildfire effect (green; Fire2-Fire2R) in the storm region over the storm periods. (*C* and *D*) are the same as (*A* and *B*), except for the total heat effect of all of the wildfires (light blue; Fire2-Fire2_NH) and the heat effect of remote wildfires (brown, Fire2-Fire2_NRH). The values over the bars in (*A* and *B*) show the ratios of each effect to the total wildfire effect. In (*C* and *D*), the value for total heat effect is its ratio to total wildfire effect, and the value for remote heat effect is its ratio to the total heat effect.

Since both the remote wildfires in the WUS and local wildfires in the CUS can affect these storms, we conducted model sensitivity tests (Fire2L and Fire2R) to understand their respective roles. Fig. 2 *A* and *B* show that remote wildfires in the WUS have a larger effect on the occurrences of heavy precipitation and hail than the local wildfires in the CUS. The remote wildfire effect contributes to 67 and 60% of the increase in the occurrences of heavy precipitation rates and SSH, respectively, which are 1.6 and 1.5 times greater than the local wildfire effect. For the accumulated precipitation, the increase by the remote and local wildfire effect is 3.4 (66% of the total wildfire effect) and 2.0 mm (42% of the total wildfire effect), respectively, with the remote wildfire effect ∼1.6 times larger than the local wildfire effect. The weaker local wildfire effect is related to reduced severity compared with fires in the WUS. Note that an interaction effect occurs when both the remote and local effects interact, so the sum of both effects is not necessarily equal to 100%. A larger contribution of the remote wildfire effect occurs in the increased frequencies of strong updrafts compared with the local wildfire effect (*SI Appendix*, Fig. S5 *C* and *D*), which can result in a larger impact on precipitation and hail.

By examining the wildfire effects on each storm (*SI Appendix*, Figs. S6 and S7), the remote wildfires gradually become stronger with time. The contribution of the remote wildfire effect is smaller than the local wildfire effect on both precipitation and hail in the first two storms (July 26 to 27) but exceeds the local wildfire effect in the latter two storms (July 28 to 29). The propagation of the meteorological variables and aerosols changed by the WUS wildfires takes time (∼2 d estimated from the AOD change in *SI Appendix*, Fig. S1), and the WUS wildfires strengthened in general from July 26 to 29.

We conducted further sensitivity tests (Fire2_NH and Fire2_NRH in Table 1) to examine the contribution of the heat and aerosol changes by wildfires, respectively. Fire2_NH excluded the sensible heat of all of the wildfires and Fire2_NRH excluded the sensible heat of remote wildfires. The results showed that the heat effect from the remote wildfires contributed to 55 and 49% of the remote wildfire effect on moderate–heavy (10 to 20 mm h^−1^) and heavy precipitation rates, respectively. This suggests that the heat and aerosol effects on precipitation from the WUS wildfires may be of a similar magnitude. For hail, the contribution of the remote heat effect is 71% for severe hail and 58% for SSH, larger than its contribution to precipitation. Nonetheless, the role of the aerosol effect from the WUS wildfires would still be significant (at least 29% for severe hail and 42% for SSH, assuming the sum of both effects is 100% by ignoring the interaction effect). Therefore, both sensible heat and aerosols from the WUS wildfires play an important role in enhancing the precipitation and hailstones of the storms in the CUS, with the sensible heat effect, which changes meteorological conditions, more significant than aerosols in enhancing severe hail and SSH.

We also examined the contribution of the sensible heat effect from all wildfires (both local and remote). Approximately 46% of the total wildfire effect on accumulated precipitation, 54% on heavy rain rates, and 46% on SSH (Fig. 2 *C* and *D*) come from the sensible heat effect of all wildfires (both local and remote). The rest comes from the wildfire aerosol effects and their interactions. Therefore, the heat effect and the aerosol effect of all wildfires have a similar magnitude. For the convective intensity, both the heat and aerosol effects of wildfires increase the frequencies of strong updrafts (*SI Appendix*, Fig. S5 *E* and *F*). Furthermore, the heat effect on precipitation and hail from remote wildfires is larger than the heat effect from local wildfires, contributing to 60 to 81% of the total heat effect of all wildfires.

### Mechanisms.

**Fig. 3.**
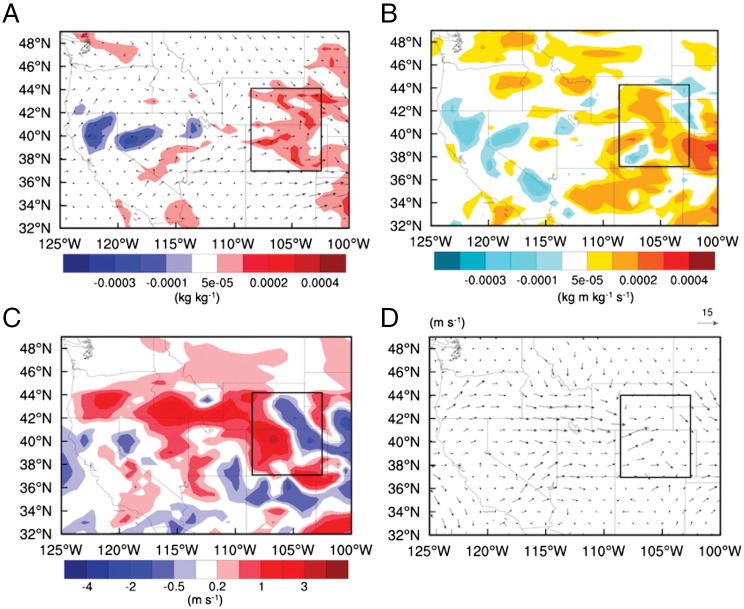
Differences in (*A*) moisture, (*B*) horizontal moisture advection, and (*C*) wind speed at 850 hPa over the storm periods between Fire1 and NoFire1. The 850-hPa wind vectors in (*A*) are averaged over the storm periods from Fire1. (*D*) The 850-hPa wind vectors for the July 29 storm from Fire1, illustrating the westerly and southwesterly winds. The black boxes denote the storm region.

To explore the mechanism responsible for the enhancement of the precipitation and hail from severe convective storms in the CUS, we examined changes in thermodynamics and dynamics over the CUS. The 2-m temperature increases by up to 2.4 °C on average from July 26 to 29 (Fig. 1*D*). The slight decrease in 2-m temperatures in the broad area is primarily attributable to the radiative effect of biomass-burning aerosols (Fig. 1*D*). During the storm periods, there is an ∼10% increase in water vapor at 850 hPa on average over the storm region in the CUS (mainly over Colorado and Wyoming; Fig. 3*A*). The increase in 2-m temperature of larger than 1 °C occurs only in the limited area in the WUS (Fig. 1*D*). The moisture increase at low levels is more significant and covers a much larger area of the CUS compared with the temperature changes (Figs. 1*D* and 3*A* and *SI Appendix*, Fig. S4*B*). Therefore, the increase in moisture in the CUS should be an important factor supporting the formation of stronger storms due to the wildfires.

**Fig. 4.**
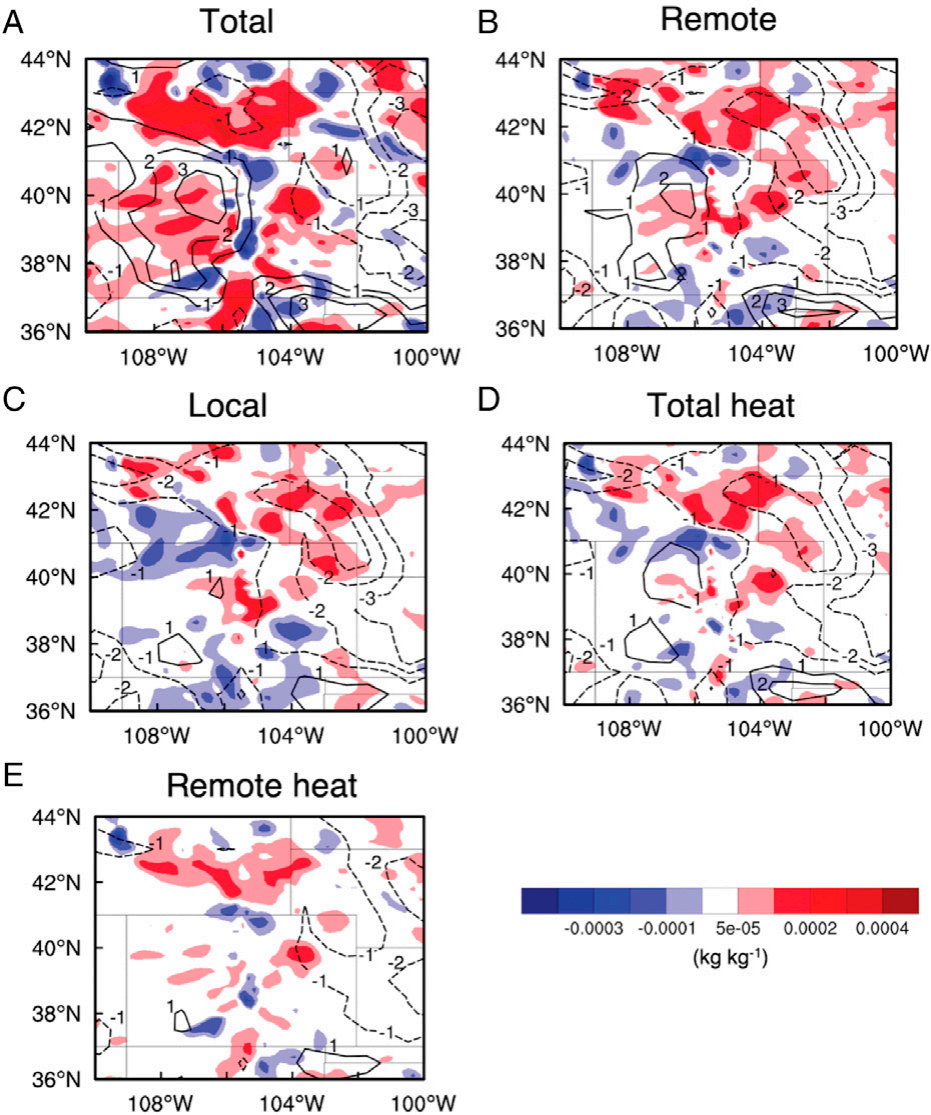
Differences in moisture (shaded color) and wind speed (contour lines) at 850 hPa in the CUS due to (*A*) total wildfire effect (Fire2-NoFire2), (*B*) remote wildfire effect (Fire2-Fire2L), (*C*) local wildfire effect (Fire2-Fire2R), (*D*) total heat effect (Fire2-Fire2_NH), and (*E*) remote heat effect (Fire2-Fire2_NRH) during the latter two storm periods. Solid (dashed) contour lines denote increased (decreased) wind speeds.

The increase in the low-level moisture in the storm region (the black box in Fig. 3*A*) is mainly due to increased moisture transport westerly and southwesterly (Fig. 3*B*). The increased moisture transport results from the intensified westerly and southwesterly winds (Fig. 3 *C* and *D*) since the water vapor content in the WUS is either reduced in the wildfire area (likely due to aerosol condensation growth and formation of pyroCb) or not changed appreciably (Fig. 3*A*). Note that the largely increased moisture transport at the east of the domain in Fig. 3*B* is not relevant to the storms being studied since it does not flow into the storm region (Fig. 3*D*). Increases in the westerly and southwesterly winds by the wildfires are at least partially associated with the increased pressure in the WUS and the decreased pressure in the CUS at low levels (e.g., 925 hPa; *SI Appendix*, Fig. S8*A*). That is, the near-surface high in the WUS increases, mainly contributed by the WUS wildfires, and the near-surface low in the CUS decreases, primarily due to the CUS wildfires (*SI Appendix*, Fig. S8 *B*–*D*). This would increase the westerly and southwesterly winds due to a stronger horizontal pressure gradient. In addition, the stronger surface low in the CUS would produce stronger convergence and storms locally, which would further strengthen the westerly and southwesterly winds in the western part of the storm region but weaken the westerly winds in the eastern part of the storm region (Fig. 4*A*). The weakened winds in the east appear in both remote and local wildfire effects (Fig. 4 *B* and *C*). It could be mainly associated with stronger storms blocking the background westerly winds.

These results indicate that wildfires change lower tropospheric dynamics and circulation (the jet stream located at the higher levels is not affected). As for reasons for the intensified surface low caused by the CUS wildfires, convective storms and precipitation occurred every day during July 26 to 29, and the stronger low anomaly could be a result of a larger removal of gaseous pollutants from wildfires by stronger precipitation since we do see decreased gaseous pollutant concentrations. Since the remote wildfire effect enhances storms but does not contribute appreciably to the stronger near-surface low, we infer that the stronger low anomaly is not related to the stronger storms.

With increased westerly and southwesterly winds and moisture in the western part of the storm region, convective available potential energy (CAPE) and convective inhibition increase at the time before each storm (*SI Appendix*, Fig. S9 *A* and *B*; each peak in the CAPE plot is at a time before a storm is initiated). The CAPE increase should be due to the increased low-level moisture since the temperature profile is not changed much. We also observed enhanced 0- to 6-km wind shear and 0- to 3-km storm-relative helicity (SRH), particularly for the latter two storms (*SI Appendix*, Fig. S9 *C* and *D*). Further analysis shows that the increases in low-level westerly winds and moisture in the western part of the storm region primarily occur in the latter two storms (Fig. 4*A*). Both the remote and local wildfire effects contribute to the increases, with the remote wildfire effect making a larger contribution (Fig. 4 *B* and *C*). This partially explains the greater impact of the remote wildfire effect on heavy precipitation and SSH compared with the local wildfire effect. Comparing Fig. 4 *D* with *A*, the total heat effect from WUS and CUS wildfires partially contributes to the stronger westerly winds and increased moisture, and a large contribution is from the aerosol effect and the interaction effect between the heat and aerosol effects. Similarly, the remote heat effect is a large contributor to the wind and moisture increase by the total heat effect (Fig. 4 *E* versus *D*).

For the wildfire aerosol effect, which has a similar magnitude as the heat effect, similarly, we see increased wind and moisture transport (the difference between Fig. 4 *A* and *D*) and increases convective intensity (*SI Appendix*, Fig. S5*F*). The wildfire aerosol number concentrations increase with the time during the simulation period (red versus blue lines in *SI Appendix*, Fig. S9*E*). The latter two storm periods exhibit a 40% increase in aerosol number concentrations compared with the first two storm periods, mainly due to the increased transport of biomass-burning aerosols from the WUS (shown from the gap between the red and green lines in *SI Appendix*, Fig. S9*E*). This partially explains the increased effect of remote wildfires with time. As for the mechanism, our previous studies with the same model have demonstrated that, for severe convective storms, increasing cloud condensation nuclei (CCN) can enhance precipitation and hail by (1) strengthening convective intensity owing to enhanced latent heat release from both warm phase and ice phase and (2) increasing supercooled cloud droplets, thus enhancing riming growth per hail embryo (16, 29). Stronger updrafts can lift and hold hail to increase residence time for growth within the updraft, although overly strong updrafts may eject hail out of the optimal growth region (31). Increasing CCN produces numerous small hail embryos that can reenter cloud updrafts and grow into larger hailstones (32, 33). More supercooled droplets allow hail embryos to reach larger sizes in the course of recycling (34, 35). In short, aerosols from wildfires play an important role in increasing the occurrences of heavy precipitation rates and SSH mainly through aerosol–cloud interactions, which also feed back to the circulation as stronger winds and transport are seen. The effect of aerosol–radiation interaction should be small as indicated by the modest change in the near-surface temperature (Fig. 1*D*), probably due to the compensation from the heat of wildfires. Our previous study on the wildfire effect also showed a small aerosol–radiation interaction effect on storm properties (16).

## Discussion

We present here a study of the impact of wildfires in the WUS (mainly California and Oregon) on severe convective storms and weather hazards in the CUS by using unique modeling developments based on the heat flux from wildfires and a spectral-bin cloud scheme for explicitly simulating aerosol–cloud interactions. Wildfires notably enhance the severity of storms in the CUS by increasing the occurrences of heavy precipitation rates (>20 mm h^−1^) and SSH (>2 in. in diameter) by 38% and 34%, respectively. The accumulated precipitation increases by ∼19%. The remote effect of wildfires in the WUS is more significant than the effect of local wildfires in the CUS (1.6 times larger in heavy precipitation rate occurrence and accumulated precipitation and 1.5 times larger in significant severe hail) because of the stronger severity of WUS wildfires. Both sensible heat and aerosols from the wildfires in the WUS play an important role in enhancing the occurrences of heavy precipitation rates and large hail in the CUS. Moreover, the heat effect may play a larger role than the aerosol effect in enhancing occurrences of large hail.

**Fig. 5.**
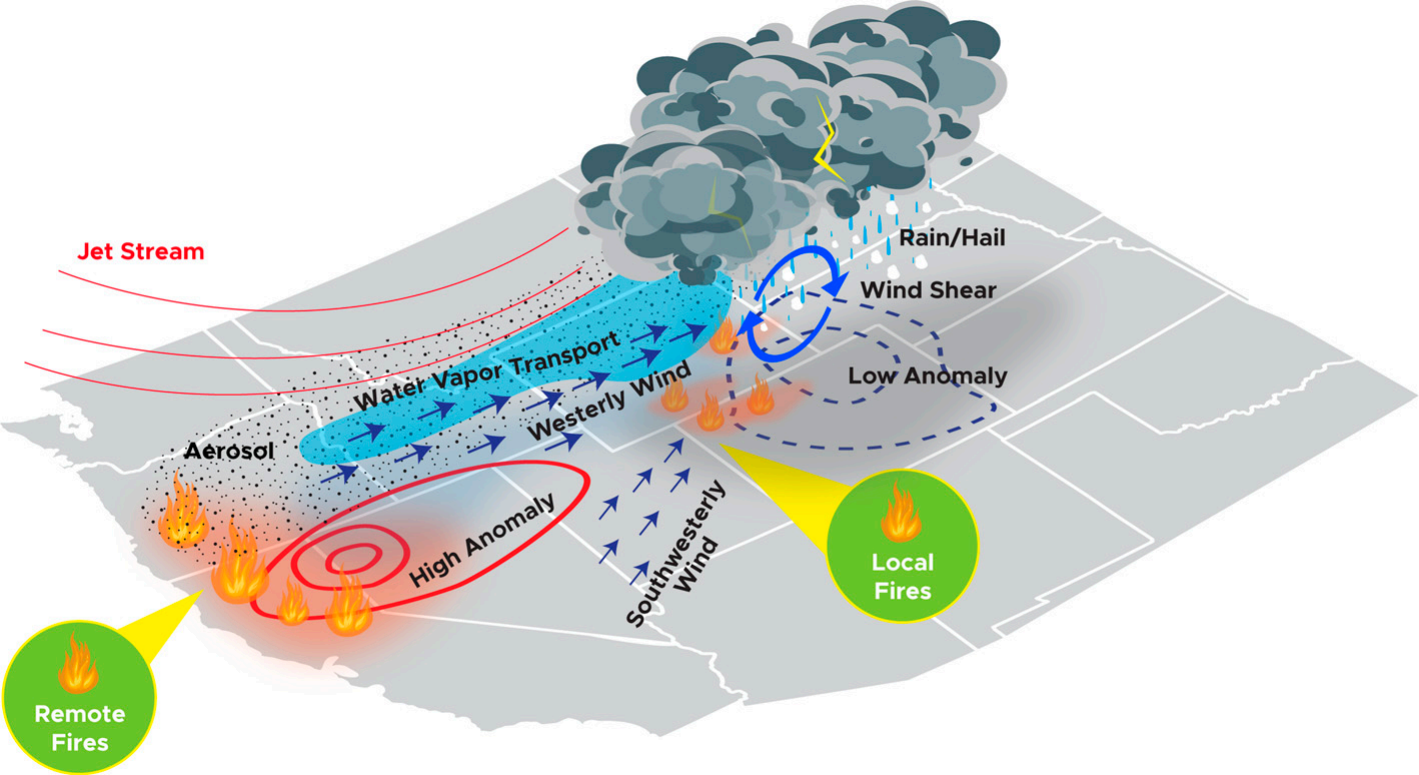
Schematic depiction of wildfire effects on severe convective storms in the CUS.

The mechanism that leads to the significant effects of wildfires on severe convective storms in the CUS is summarized in Fig. 5. Wildfires enhance surface high pressure in the WUS and the surface low pressure in the CUS and increase westerly and southwesterly winds. This leads to (1) stronger moisture and aerosol transport to the CUS and (2) larger wind shear and SRH in the CUS. Both the meteorological environment that is more conducive to severe convective storms and increased aerosols contribute to the increased occurrences of heavy precipitation rates and large hail. The remote wildfires produce larger changes in both meteorology and aerosols than the local wildfires, which are less severe compared to the remote fires, thus producing a larger effect. The effect of remote wildfires becomes larger as time progresses because the transport of moisture and aerosols strengthen with time.

This study suggests a pathway to changing severe convective storms and weather hazards in the CUS by upstream wildfires. This concept may apply to other regions influenced by upstream fires. Because the remote wildfire effects revealed here occur through enhancing the high surface pressure in the WUS, which is the general condition for wildfire weather, and the low surface pressure in the CUS, which is also the general condition for severe convective storms, we argue that the results can be qualitatively generalized to any event of this kind. The future projection in a warming climate showed that both the wildfire potential in the WUS and severe weather in the CUS in summer would be increased (24, 25). Therefore, it is reasonable to expect that such co-occurring events occur more frequently and the impact of WUS wildfires on the CUS storms may become increasingly important in the future as climate warming continues. Finally, we note that global climate models (GCMs) are not able to consider the wildfire effects examined here because deep convective storms and the sensible heat of wildfires are subgrid processes that are not explicitly represented in GCMs.

## Materials and Methods

### Wildfires and Storms.

The large and destructive wildfire events in the WUS during the 1-wk period of July 23 to 29, 2018 were located mainly in northern California and southern Oregon (*SI Appendix*, Fig. S2*A*). These events include the Carr Fire (started on July 23 and burned >450 km^2^ by July 31), the Mendocino Complex Fire (started on July 27 and burned >300 km^2^ by July 31), the Long Hollow Fire (started on July 26 and burned >130 km^2^ by July 31), the Whaleback Fire (started on July 27 and burned ∼70 km^2^ by July 31), and the Cranston Fire (started on July 26 and burned ∼50 km^2^ by July 31). Some of those fires, such as the Carr Fire, produced pyroCbs (36). The wildfires in the WUS strengthened over the 1-week period of July 23 to 29. In the CUS, there were also several fires (*SI Appendix*, Fig. S2*A*), such as the Grand Teton Fire (started on July 24), the Plateau Fire (started on July 22), and the Bull Draw Fire (started on July 29, smaller than those occurring in the WUS, with a burned area of each fire of no more than 20 km^2^ by July 31).

During July 26 to 29, 2018, the sensible heat fluxes from the wildfires averaged over the fire-burned area have a range of 45 to 80 kW m^−2^ in WUS and 8 to 35 kW m^−2^ in CUS based on the satellite observations. It is 2 to 3 orders of magnitude larger than the background sensible heat flux over July, which has a range of −0.05 to 0.5 kW m^−2^, and no significant difference between WUS and CUS.

Among the four storms during July 26 to 29, the July 29 supercell thunderstorm was the strongest, causing widespread damage over eastern Colorado (see the National Weather Service report at https://www.weather.gov/gld/July292019SevereStorms). The observed hail was up to baseball size and straight-line gusting winds were between 75 and 105 mph.

### Simulations and Analysis.

Two nested domains with horizontal grid spacings of 3 and 1 km and 65 vertical levels were used (*SI Appendix*, Fig. S1*A*). Domain 1 (D01) covers both the WUS and CUS. Domain 2 (D02), which covers the CUS only, was run separately from D01 using the nest down approach, with the initial and lateral boundary conditions of gases, aerosols, and meteorology from D01. This setup allowed us to examine the effects of wildfires in the WUS separately (i.e., by changing the initial and lateral boundary conditions of meteorology and aerosols for D02). Model simulations over D02 using WRF-Chem-SBM version 3.9.1 based on Gao et al. (27) were coupled with the development of incorporating the heat flux of wildfire in Zhang et al. (16). The aerosol module used is the model for simulating aerosol interactions and chemistry (MOSAIC), with four bins (37). For D01 simulations, the Morrison 2-moment bulk scheme (38) with the hail option was used to save computation costs since the purpose of D01 simulations is to provide the initial and boundary layer conditions, and limited clouds exist outside of the CUS. The treatment of heat fluxes in the model was described in the earlier study by Zhang et al. (16). All of the wildfires were aggregated into three categories (forest, woody savanna, and grassland) based on the land use dataset. The heat fluxes used for the forest, woody savanna, and grassland were 80, 23, and 3.3 kW m^−2^, respectively, based on Freitas et al. (39). The location and time of the fires were identified globally using the MODIS thermal anomalies product. At each grid, the vegetation types of wildfires and their corresponding burned area were identified with the MODIS vegetation continuous fields product. The heat flux from the wildfires was calculated by the unit heat flux of each vegetation type multiplied by the corresponding burned area. Since the burned area can be smaller than the area of the grid box, the grid-mean heat flux was obtained by further being divided by the grid box area. Vertically, the heat flux was assumed to be an exponential decay from the surface to the fire plume height (40). The grid-scale heat flux was then treated as an additional forcing term in the thermodynamics equation. Temporally, the heat flux was applied to the whole day if the day was a fire day since we did not have the diurnal information that would produce some model uncertainty. Zhang et al. (16) applied this method in WRF-Chem to investigate the impact of wildfires on thermodynamics and severe convective storms. Incorporating heat flux to the lower atmosphere into regional and global climate models is often used to investigate the influence on regional weather and climate, such as the impact of anthropogenic heat as in Block et al. (41) and Flanner (42). The temperature increase in the fire area shown in Fig. 1*D* is consistent with the studies in the literature (40).

Other physics schemes applied to all of the simulations include the Unified Noah land surface scheme (43), the Yonsei University planetary boundary layer scheme (44), the rapid radiative transfer model for general circulation model longwave, and shortwave radiation schemes (45). The anthropogenic emissions were from NEI-2011 emissions. Biogenic emissions are represented by the model of emissions of gases and aerosols from nature (MEGAN) product (46). Biomass burning emissions from the Quick Fire Emission Dataset (QFED) emissions version 2.5 were used (47). QFED has particulate emissions including primary organic aerosol, black carbon, PM2.5, and trace gas emissions, including CO, NH_3_, NO, and SO_2_, and nonmethane volatile organic compounds.

Meteorological initial and lateral boundary conditions for D01 were produced from the Rapid Refresh model, which primarily comprises a numerical forecast model and an analysis/assimilation system at a 13-km resolution (48). The chemical initial and lateral boundary conditions for D01 were created from the Modern-Era Retrospective Analysis for Research and Applications, version 2 (MERRA-2) (49). The D01 and D02 simulations ran from 0000 UTC July 23 and 1200 UTC July 26, respectively, both ending at 0600 UTC July 30. 2018. Table 1 summarizes the simulations for this study. Fire1 and NoFire1 were the simulations over D01 covering both the WUS and CUS, with wildfire effects considered and excluded, respectively. Six simulations were run over the CUS (D02). Fire2 is the baseline simulation, with both remote wildfires in the WUS and local wildfires in the CUS considered, whereas in NoFire2 the wildfire effect (both remote and local wildfires) was excluded. The total wildfire effect was obtained from the difference between Fire2 and NoFire2. Fire2R and Fire2L were run for singling out the remote and local wildfire effects. Fire2R was a simulation with the remote wildfire effect considered but the local wildfire effect excluded, while Fire2L was the simulation with the local wildfire effect considered but the remote wildfire effect excluded. Therefore, the remote wildfire effect is the difference between Fire2 and Fire2L, and the local wildfire effect is the difference between Fire2 and Fire2R. Fire2_NH and Fire2_NRH are the sensitivity tests for examining the total heat effect and the heat effect from the remote wildfires, respectively. Fire2_NH is based on Fire2, considering the wildfire aerosol effect only (i.e., the heat effect from both remote and local wildfires was excluded). Fire2_NRH is also based on Fire2 but excludes the heat effect from the remote wildfires. The difference between Fire2 and Fire2_NH is the total heat effect of wildfires and that between Fire2 and Fire2_NRH is the heat effect from the remote wildfires.

For storm-related analysis, we focus on the storm region that is denoted by the black box in Fig. 1*C*. A storm period from each of the four storms is selected. The storm periods for July 26 and 27 are 1800 UTC July 26 to 0200 UTC July 27 and 2000 UTC July 27 to 0100 UTC July 28, respectively. For July 28 and 29, they are 2100 UTC July 28 to 0300 UTC July 29 and 1800 UTC July 29 to 0400 UTC July 30, respectively.

### Data for Model Evaluation.

Maximum hail sizes from the simulations were estimated using a physical-based hail forecasting model (HAILCAST) (50), which is online coupled with WRF-Chem simulations. HAILCAST forecasts the maximum expected hail diameter at the surface using updraft and microphysical information produced by WRF-Chem. For this study, we incorporated the updated HAILCAST version from WRF version 4.0 (51) into the WRF-Chem version 3.9.1. Two observational datasets were used for hail evaluation: the NOAA Storm Prediction Center (SPC) report (same data for hail as the National Centers for Environmental Information [NCEI] database) and the maximum expected size of hail (MESH) data. The MESH data used in this study were developed from a newly-improved algorithm (52). The large differences between the SPC and MESH datasets as shown in Fig. 1 *E* and *F* indicate a large uncertainty with the observed maximum hail sizes. The evaluation of PM2.5 uses the surface daily data from EPA (https://www.epa.gov/outdoor-air-quality-data/download-daily-data). The smoke plume height data digitized from MISR based on the MISR Interactive Explorer software were used to evaluate the predicted plume height. The National Centers for Environmental Prediction/Environmental Modeling Center Stage IV Data were used for the observation of precipitation at a 4-km resolution (53).

### Observational Analysis of Concurrences of Wildfires and Storms.

We examined the events with co-occurrences of storms in the CUS and wildfires in the WUS over 10 y, from 2009 to 2018. In this analysis, the WUS states include California, Oregon, and Washington and the CUS states include Montana, Wyoming, Colorado, New Mexico, North Dakota, South Dakota, Nebraska, Kansas, Oklahoma, and Texas. We used the daily wildfires from the Fire Program Analysis Fire-Occurrence Database (54) and the storm event data from the NCEI (www.ncdc.noaa.gov/stormevents). We first paired the daily fire-burned area with the storm events with daily hail and heavy rain reports in the CUS for the warm season from March to August to find the co-occurrence events. Then, to consider the time lag for the remote wildfire effect, we added a condition for the wildfires; that is, wildfires occur not only in the present day with severe weather events but also in the previous 2 d. For example, for a selected storm occurring on July 26, not only 26 but also 24 and 25 must be fire days. Only storm events exceeding 20 reports for the sum of hail and heavy rain and the wildfire events with a burned area >20 km^2^ are sufficiently significant to be considered. We recorded the storm event with consecutive days, for example, 1, 2, 3, and 4 consecutive days. Four consecutive day events means that storms are occurring 4 d in a row.

## Supplementary Material

Supplementary File

## Data Availability

Simulations data have been deposited in https://portal.nersc.gov/project/m2977/RemoteFireEffect (55).
