# Supplementary material for: Notable impact of wildfires in the western United States on weather hazards in the central United States
Source: Proc Natl Acad Sci U S A. 2022 Oct 17;119(44):e2207329119. doi: 10.1073/pnas.2207329119 (PMC9636965; doi:10.1073/pnas.2207329119)
Supplement: Supplementary File [file pnas.2207329119.sapp.pdf]

PNAS
www.pnas.org

Supplementary Information for

Notable Impacts of Wildfires in the Western US on Weather Hazards in the Central US

Yuwei Zhang, Jiwen Fan*, Manish Shrivastava, Cameron R. Homeyer, Yuan Wang, John Seinfeld

*Corresponding author: Jiwen Fan

Email: jiwen.fan@pnnl.gov

This PDF file includes:

Figures S1 to S9

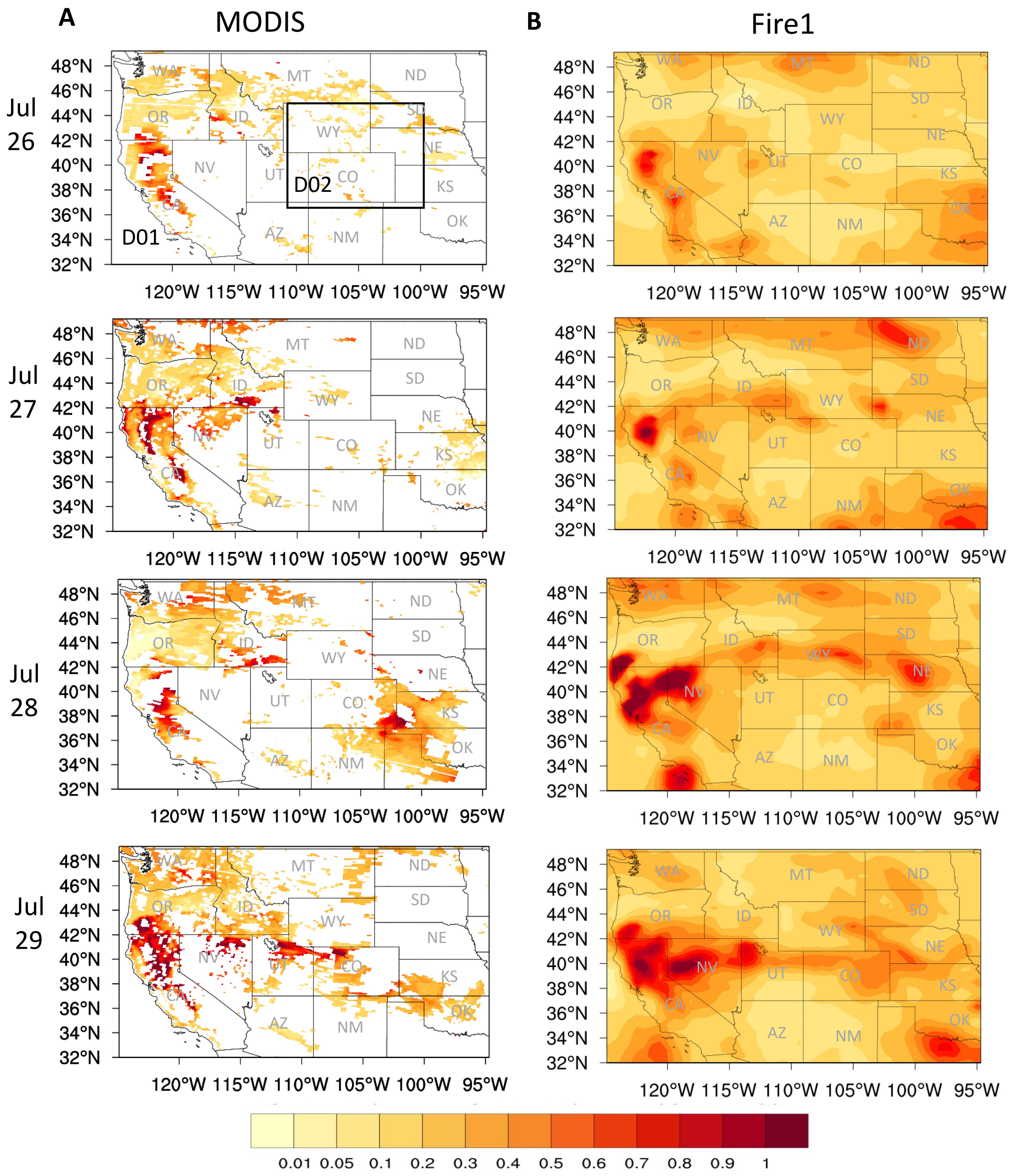

Fig. S1. AOD from (A) MODIS (left) and (B) Fire1 (right) from 26 to 29 July 2018. The two model domains are shown as D01 and D02 in (A).

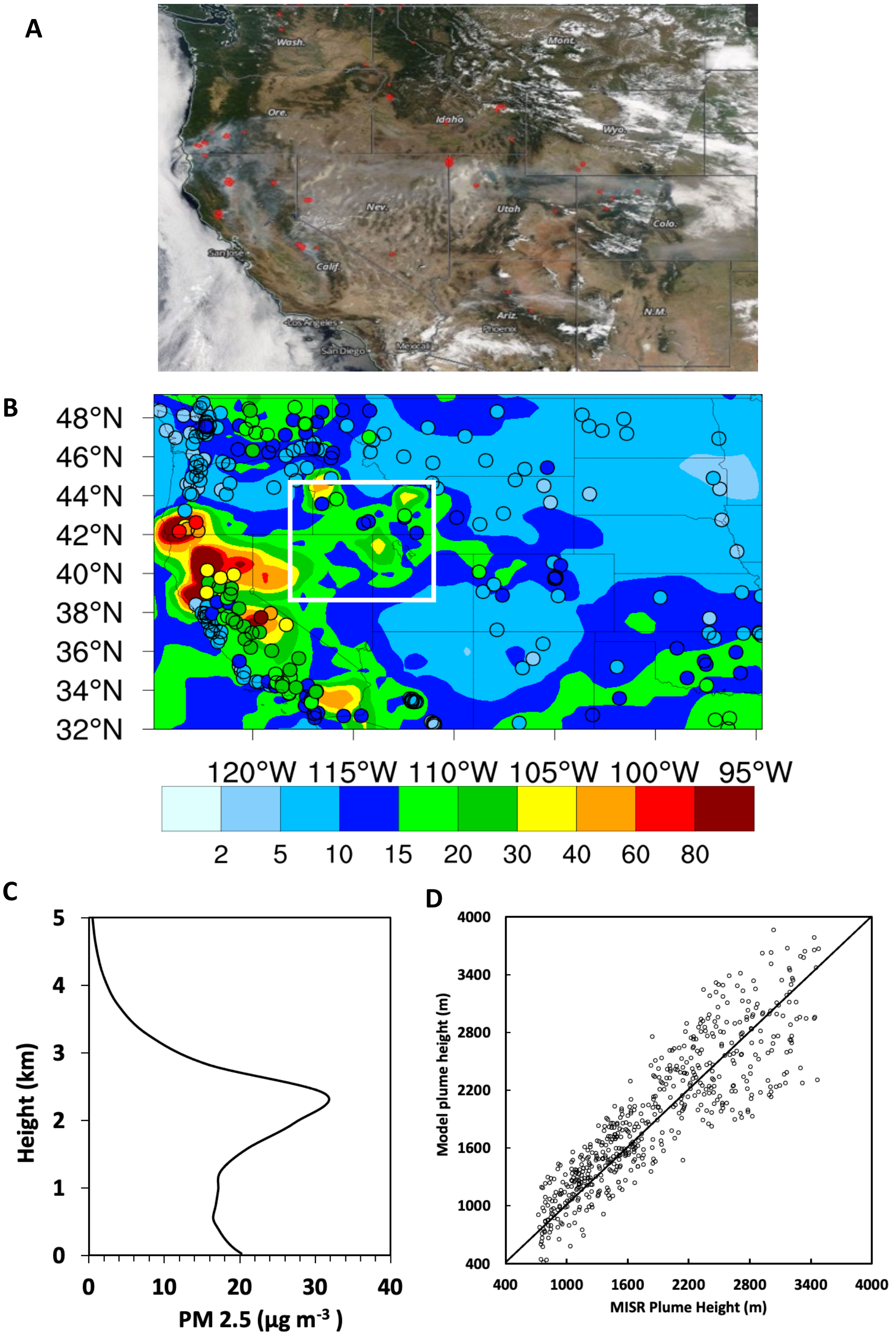

Fig. S2. (A) Wildfire locations based on the fire and thermal anomalies detected from the MODIS on 29 July. The red dots represent wildfire locations. The plot is produced from https://worldview.earthdata.nasa.gov/. (B) Surface PM2.5 from Fire1 (color contours) and surface observations (colored circles) from EPA averaged over 26-29 July 2018. (C) Vertical distribution of PM 2.5 averaged over the white box in (B) during 26-29 July. (D)Comparison of plume heights between MISR and Fire1 during 1900-1930 UTC on 27 July and 1830-1900 UTC on 29 July for the WUS wildfires.

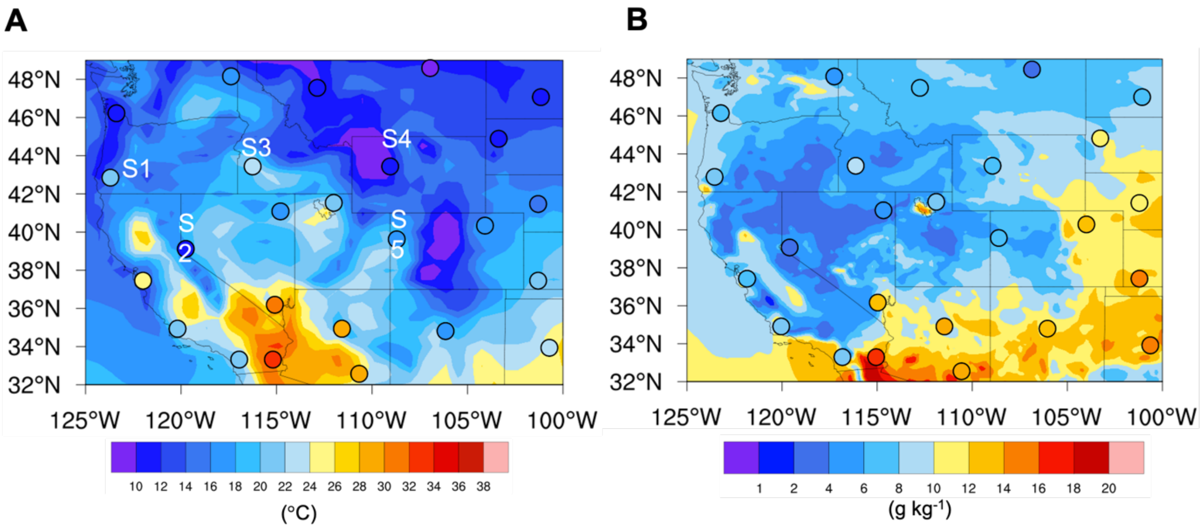

Fig. S3. 2-m (A) temperature and (B) water vapor mixing ratio from Fire1 (color contours) and sounding data (colored circles). The simulation and observation are averaged over the times of sounding data during 26-29 July 2018. S1, S2, S3, S4, and S5 in (A) mark the sounding sites near wildfires that are further examined in fig. S4.

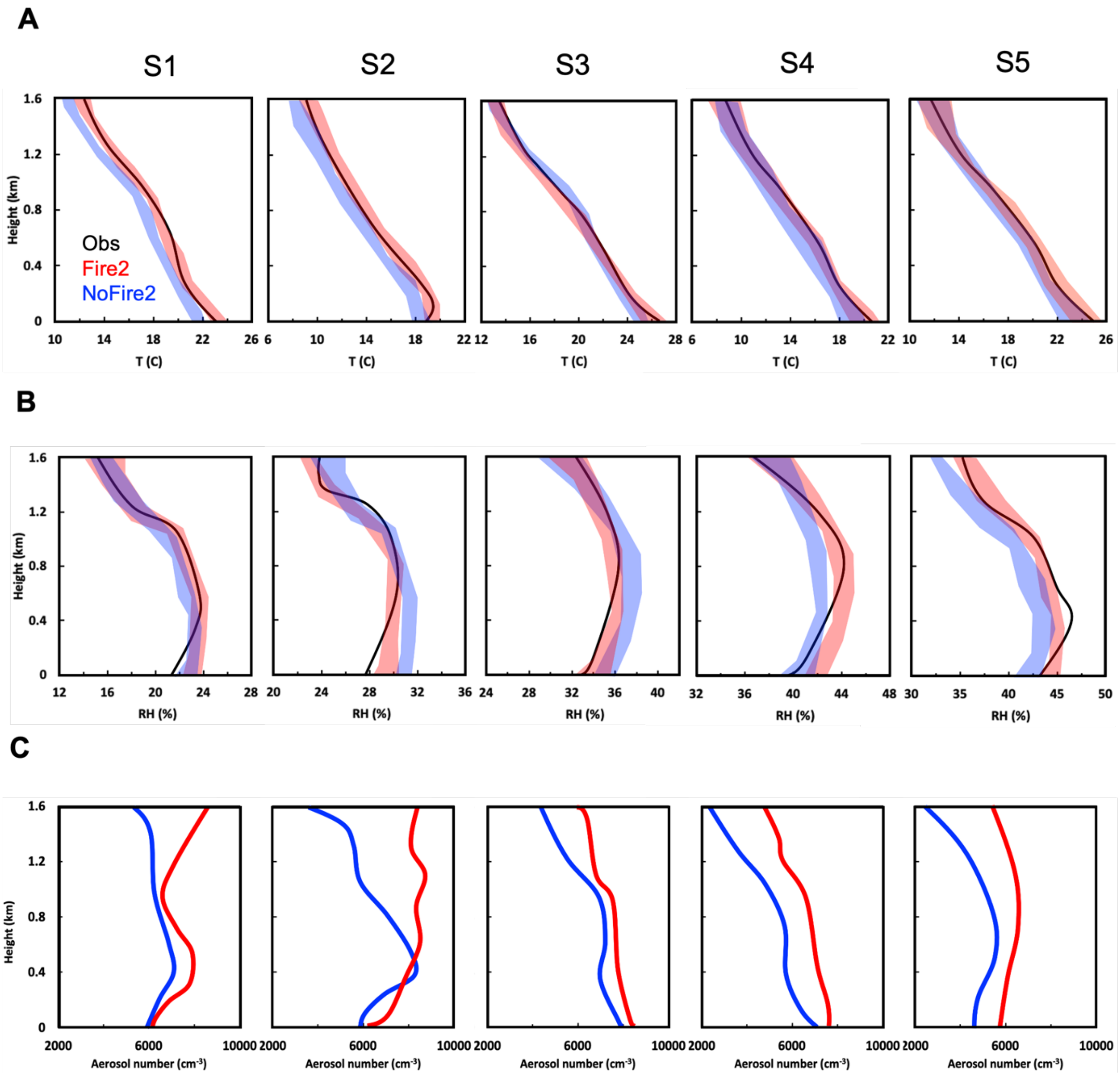

Fig. S4. 2-m (A) temperature, (B) RH, and (C) aerosol number concentration of five sounding sites marked in fig. S2A from observations (black), Fire2 (red), and NoFire2 (blue) averaged over the observation times of soundings (twice a day) during 26-29 July 2018. Shaded denoted the variation range of the model data (i.e., from minimum to maximum) within the 100 km^2 areas of the sounding site.

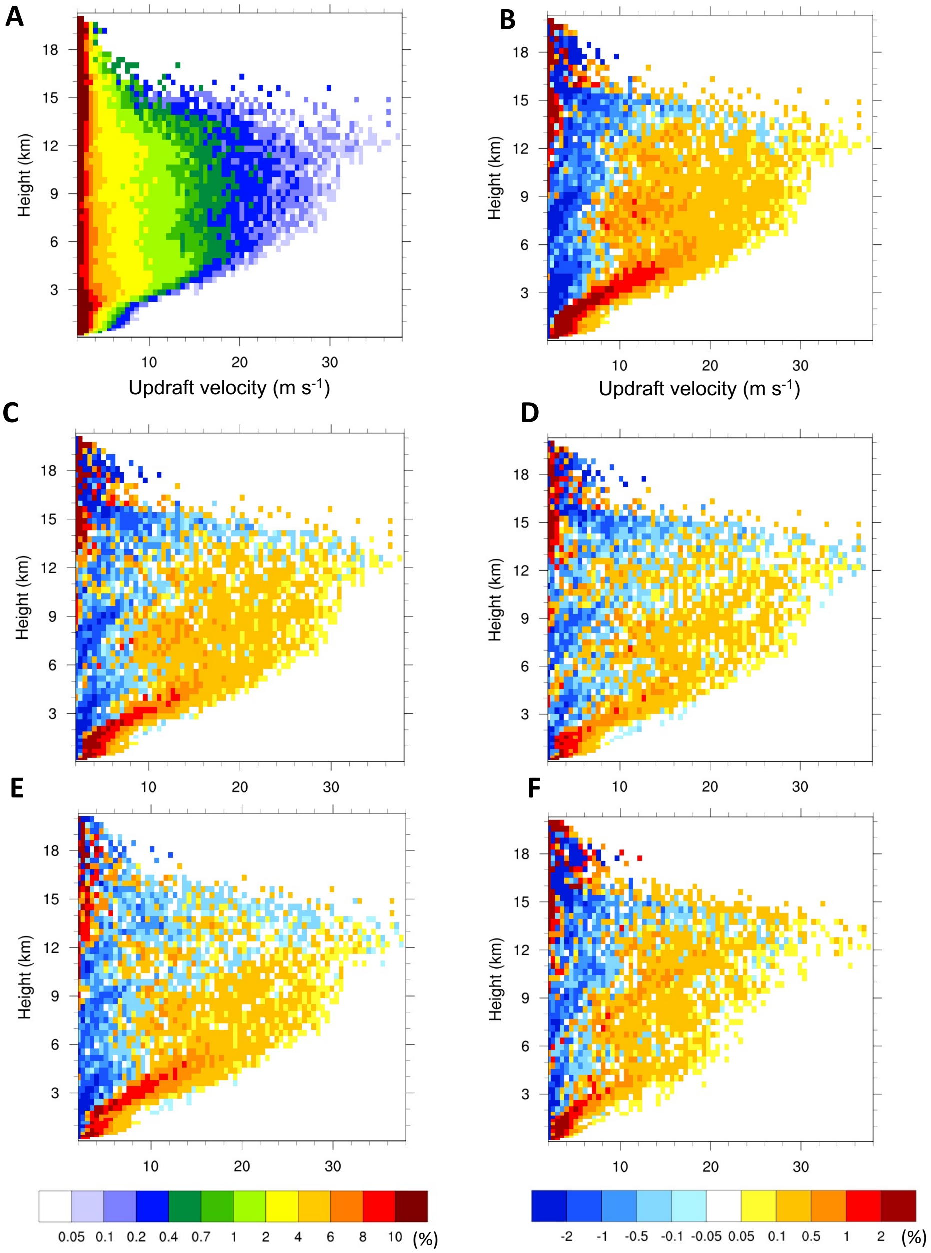

Fig. S5. (A) Normalized frequency distribution of updraft velocity larger than 2 m s^{-1} over the vertical profiles for Fire2 in the storm region during the storm periods. Panels (B)-(F) present the differences in normalized frequency distribution for (B) total wildfire effect (Fire2-NoFire2), (C) remote wildfire effect (Fire2-Fire2L), (D) local wildfire effect (Fire2-Fire2R), (E) wildfire heat effect (Fire2-Fire2_NH), and (F) wildfire aerosol effect (Fire2_NH-NoFire2).

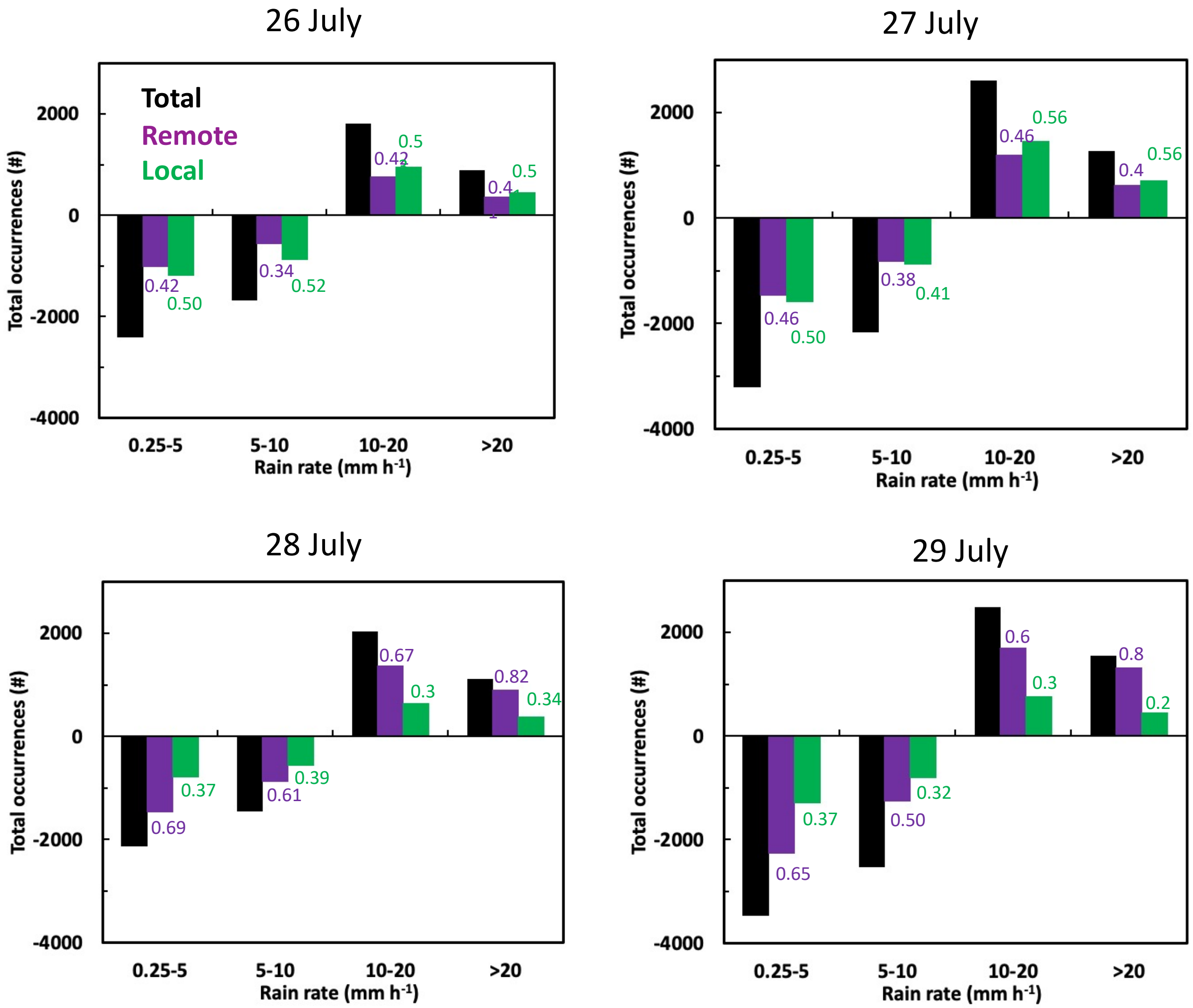

Fig. S6. Differences in occurrences of rain rates of 0.25-5, 5-10, 10-20, and >20 mm h^{-1} due to the total wildfire effect (black; Fire2-NoFire2), remote wildfire effect (purple; Fire2-Fire2L), and local wildfire effect (green; Fire2-Fire2R) in the storm region over the storm period for each storm. The value on the bar is the ratio of each effect to the total wildfire effect.

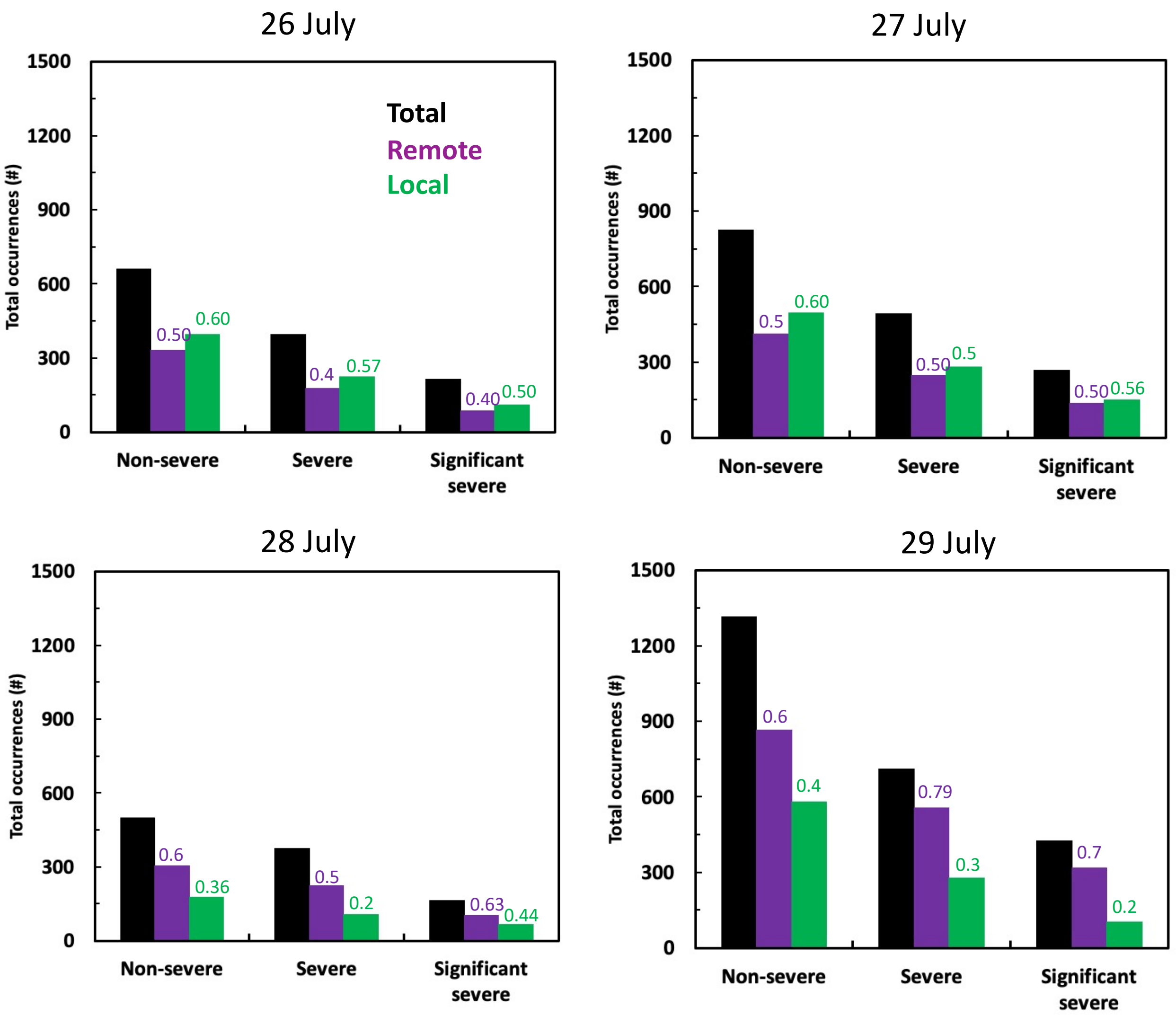

Fig. S7. Same as fig. S6, except for non-severe (0.2 ≤ diameter < 1 inch), severe (1 ≤ diameter < 2 inches), and significant severe hail (2.5 inches or larger).

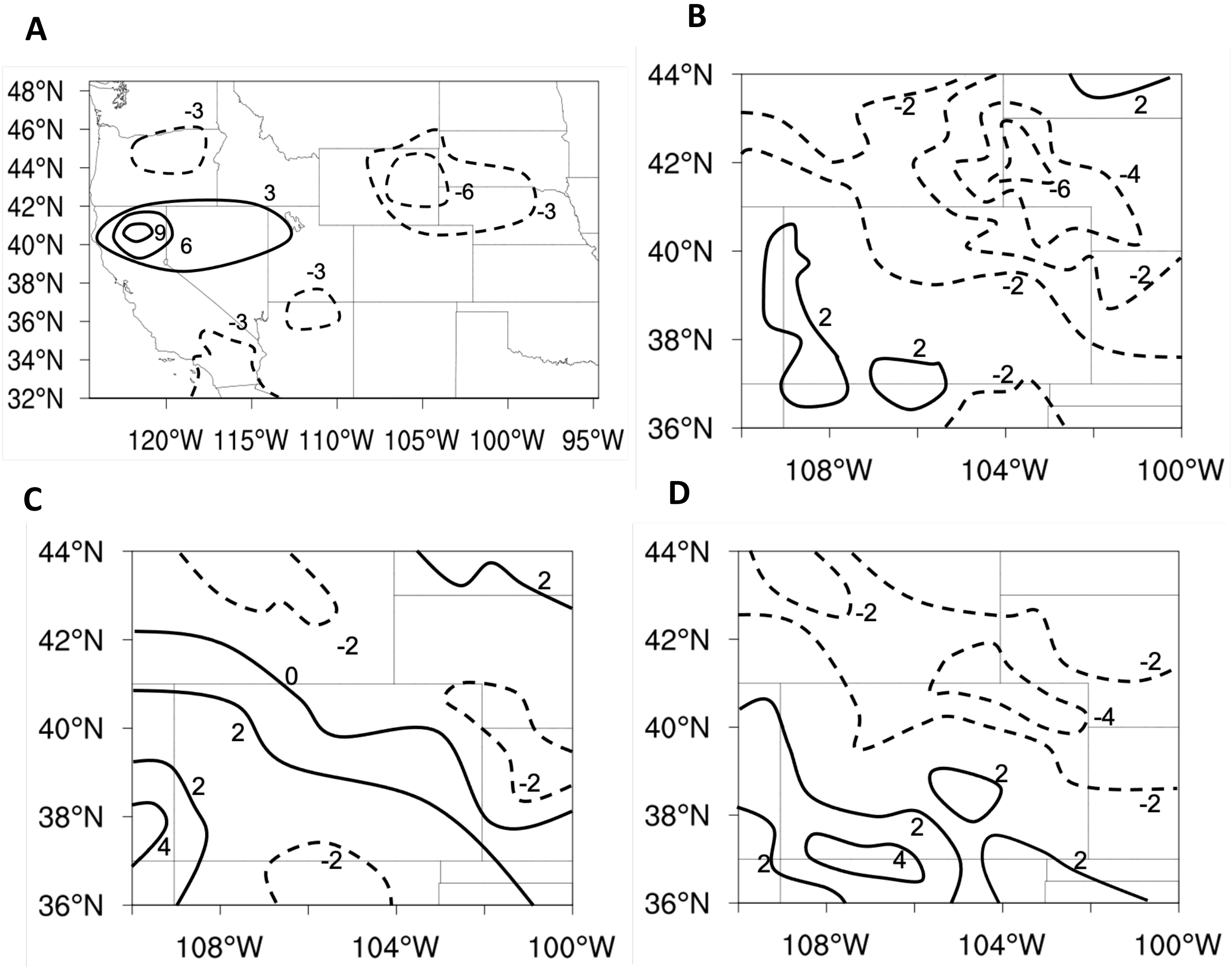

Fig. S8. Differences in geopotential height at 925 hPa averaged over the latter two storm periods (A) due to the wildfire effect (Fire1 - NoFire1) in both the WUS and CUS. (B) the same as (A), except for the CUS only from 1-km simulations (i.e., Fire2-NoFire2). (C-D) same as (B), except for the remote wildfire effect and local wildfire effect, respectively. Solid (dashed) contour lines denote positive (negative) anomalies.

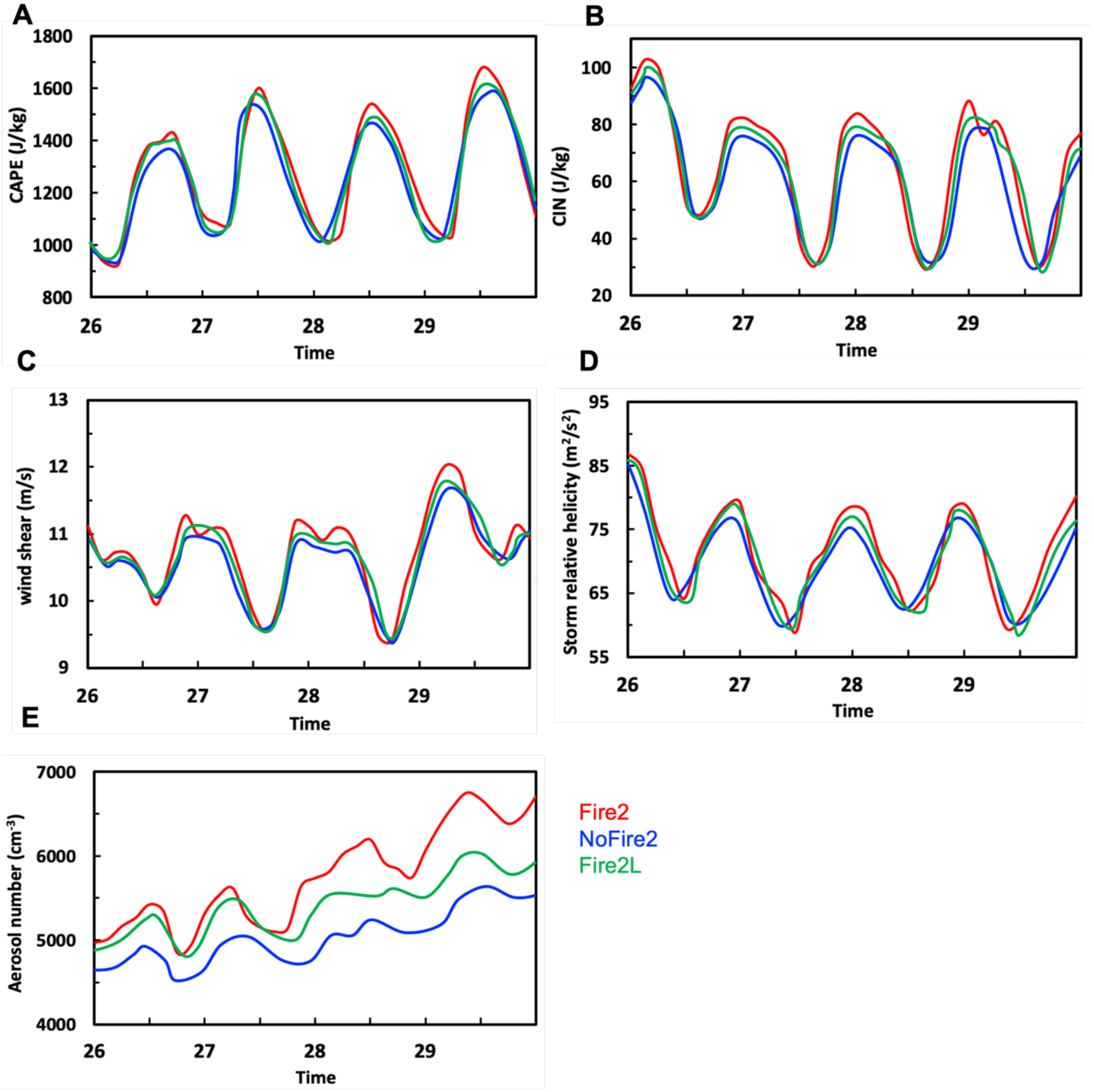

Fig. S9. Time series of (A) convective available potential energy (CAPE), (B) convective inhibition (CIN), (C) 0-6 km wind shear, (D) 0-3 km storm-relative helicity (SRH), and (E) aerosol number concentration for Fire2 (red), NoFire2 (blue), and Fire2L (green) at the storm region during 26-29 July.
